# Supplementary material for: A penalized variable selection ensemble algorithm for high-dimensional group-structured data
Source: PLoS One. 2024 Feb 5;19(2):e0296748. doi: 10.1371/journal.pone.0296748 (PMC10843621; doi:10.1371/journal.pone.0296748)
Supplement: Attachment — Submitted filename: Response to Reviewers.docx [file pone.0296748.s002.docx]

Author's Response to Reviewer Comments

PONE-D-23-35145

Dear Editors and Reviewers,

We highly appreciate the Reviewer’s efforts and time in reviewing our manuscript entitled “**A Penalized Variable Selection Ensemble Algorithm for High-Dimensional Group-Structured Data**”(**Manuscript ID:** **PONE-D-23-35145**) for **PLOS ONE**. The Reviewers’ comments are positive and constructive, which are very valuable and helpful for revising and improving our paper, as well as for improving our future research. Based on the Reviewer’s suggestions, we have made corresponding revisions highlighted in red over the whole manuscript to clarify the points of the reviewers.

Here are our point-by-point responses to the Editors and Reviewers’ comments.

Editors’ Comments

**(1)** Please ensure that your manuscript meets PLOS ONE's style requirements, including those for file naming.

**[Authors’ Reply]:** Many thanks for your kind help in dealing with our paper. In this revision, We have made changes to the title font size, author information and unit information, and corresponding authors involved in this paper according to the requirements of the journal.

**(2)** Please note that PLOS ONE has specific guidelines on code sharing for submissions in which author-generated code underpins the findings in the manuscript. In these cases, all author-generated code must be made available without restrictions upon publication of the work.

**[Authors’ Reply]:** Many thanks for your kind help in dealing with our paper. In this revision, We have organized the main code of the paper and made it available upon request, ensuring that the code is shared in a way that follows best practices and promotes reproducibility and reuse.

**(3)** Thank you for stating in your Funding Statement:

“This work was supported by The Guizhou Provincial Department of Education's Youth Growth Project Fund [No. Qian Jiaoji [2022] 380, No. Qian Jiaoji [2022] 377, No. Qian Jiaoji [2022] 378, and No. Qian Jiaohe KY [2021] 282]; and in part by the Educational Department of Guizhou under Grant [No.KY[2019]067].”

Please provide an amended statement that declares *all* the funding or sources of support (whether external or internal to your organization) received during this study, as detailed online in our guide for authors at http://journals.plos.org/plosone/s/submit-now. Please also include the statement “There was no additional external funding received for this study.” in your updated Funding Statement.

**[Authors’ Reply]:** Many thanks for your kind help in dealing with our paper. In this revision, We describe all funds received for this study here and supported by all funds.

**Funding：**

This work was supported by The Guizhou Provincial Department of Education's Youth Growth Project Fund [No. Qian Jiaoji [2022] 380, No. Qian Jiaoji [2022] 377, No. Qian Jiaoji [2022] 378, and No. Qian Jiaohe KY [2021] 282]; and in part by the Educational Department of Guizhou under Grant [No.KY[2019]067].

**Competing interests**

The authors have declared that no competing interests exist.

**(4)** We note that the grant information you provided in the ‘Funding Information’ and ‘Financial Disclosure’ sections does not match.

**[Authors’ Reply]:** Many thanks for your kind help in dealing with our paper. In this revision, As we resubmit, we will note the issue of the 'Funding Information' and 'Financial Disclosure' sections do not match and make the corrections as required.

**(5)** We note that you have provided additional information within the Acknowledgements Section that is not currently declared in your Funding Statement. Please note that funding information should not appear in the Acknowledgments section or other areas of your manuscript. We will only publish funding information present in the Funding Statement section of the online submission form.

When you resubmit, please ensure that you provide the correct grant numbers for the awards you received for your study in the ‘Funding Information’ section.

Please include your amended Funding Statement within your cover letter. We will change the online submission form on your behalf.

**[Authors’ Reply]:** Many thanks for your kind help in dealing with our paper. In this revision, We have removed the information about the grant-related information from the text and provided information about the grant funds for this research in the cover letter, thank you for the advice you provided editor, and please help us to update the grant statement.

**(6)** Please review your reference list to ensure that it is complete and correct. If you have cited papers that have been retracted, please include the rationale for doing so in the manuscript text, or remove these references and replace them with relevant current references. Any changes to the reference list should be mentioned in the rebuttal letter that accompanies your revised manuscript. If you need to cite a retracted article, indicate the article’s retracted status in the References list and also include a citation and full reference for the retraction notice.

**[Authors’ Reply]:** Many thanks for your kind help in dealing with our paper. In this revision, We carefully reviewed the reference list and updated it as necessary to ensure its accuracy. In the paper, the author of the cited document explains and retracts the published paper due to its great similarity to the previously published article after publication. So, thank you very much for the editor's reminder, we have updated the corresponding literature. That is why the retracted literature citation

was made, and I have updated the literature to avoid unnecessary hassle.

Thanks to your care and feedback, the literature we replaced is listed below:

Original Paper:

[27]	Freund Y,Schapire E R. Game theory, on-line prediction and boosting[P]. Computational learning theory,1996.

[34]	He H, Guo X, Yu J, et al. Retracted: Overcoming the inadaptability of sparse group lasso for data with various group structures by stacking[J]. Bioinformatics, 2022, 38(6): 1542-1549.

Updated as:

[27]Schapire R E , Freund Y , Bartlett P ,et al.Boosting the margin: a new explanation for the effectiveness of voting methods[J].The Annals of Statistics, 1998, 26(5):1651-1686.

[34] Yu B, Wang X, Zhang Y, et al. RPI-MDLStack: Predicting RNA–protein interactions through deep learning with stacking strategy and LASSO[J]. Applied Soft Computing, 2022, 120: 108676.

We highly appreciate the editor’s comments about our manuscript!

Response to Reviewer:

Reviewer #1: **(1)** Your methodology, involving comprehensive simulation experiments comparing the StackingGroup model with various algorithms under different group variable scenarios, is commendable. However, for improved reader comprehension, providing context for the evaluation metrics (R², MAE, RMSE) is advised. **(2)** Additionally, a deeper explanation of data generation and the unique contributions of StackingGroup in various scenarios, along with the incorporation of visual aids, will enhance the presentation's clarity and value. **(3)** Furthermore, linking the results to practical implications for researchers and practitioners would enrich the paper's real-world relevance.

**[Authors’ Reply]:** Many thanks for your kind help in dealing with our paper. In this revision, We have added the context as well as the meaning of these three assessment indicators as follows:

**(1)** In regression problems, the three evaluation methods of *R*^2^, *MAE*, and *RMSE* are often used to evaluate the goodness of a regression model. Using these evaluation indexes can assess the performance of the model more comprehensively and objectively, and provide stronger support for the improvement and optimization of the model, each of the three evaluation methods reflects the performance of the model from a different perspective, for example, *R*^2^ measures the degree of fit of the model to the observed data, the *MAE* measures the mean absolute error between the predicted and observed values, and *RMSE* measures the root mean square error between the predicted and observed values, by using these three evaluation metrics can be corroborated with each other to mutually verify the performance of the model.

**(2)**In the simulation experiment, the variance setting for generating random error data was supplemented for the data generation paper which has been described in great detail and was supplemented as: $\sigma^{2}=10$; See Example1- Example5 in the paper for the generation of the data.

**(3)**The StackingGroup algorithm is mainly oriented to the variable selection problem with high-dimensional group structure data and proposed, in practical applications, some data with group structure, for example, an enterprise financial data according to the profitability, growth, solvency, dividend capacity, etc. will be divided into several groups of variables, each group contains a number of variables. In this case, the use of univariate selection methods will ignore the grouping information, which may lead to a significant reduction in the effectiveness of variable selection. In order to solve this problem, many scholars have proposed group structure algorithms, but they still have their own drawbacks, such as the estimation is biased, can only deal with highly correlated group selection, etc., so how to overcome the shortcomings of these algorithms? Our idea is to design an integrated learning model to solve this problem, in the process of solving this problem, we finally choose the Stacking algorithm, which can enhance the model prediction accuracy and generalization ability by multi-layer stacking through the differences of heterogeneous algorithms. The StackingGroup algorithm proposed in this paper is constructed with grSubset+grLasso, grLasso, and grSCAD algorithms as the base learner and lasso as the meta-learner. Integrated learning can solve the inherent defects of a single model or a model with a certain set of parameters, so as to integrate more models, complement the strengths of the models, and avoid limitations. The proposed algorithm can solve the problem of selecting variables with high-dimensional group structure in practice, such as in the financial field, the algorithm can be applied to bond rating and portfolio optimization. It can help to solve the problem of variable selection with high-dimensional cluster structure data by selecting variables with high-dimensional cluster structure to evaluate the credit rating and risk status of bonds and optimize investment portfolios to improve the return of capital and the effect of risk control, especially in the field of genome and the field of medical treatment.

Reviewer: 2

**(1)** While the paper presents a novel algorithm, it would be beneficial to discuss the limitations and potential challenges associated with implementing the StackingGroup algorithm. Addressing practical issues or constraints in real-world applications would provide a more comprehensive perspective.

**[Authors’ Reply]:** Many thanks for your kind help in dealing with our paper, In this revision.

Algorithmic limitations:

In this paper, in order to explore the effect of different penalty functions on variable selection for high-dimensional group data, the experimental design only considered setting different dimensions p, 500, 1000, 1500, and 2000, without considering the sample size n. In this experiment, the sample size is taken to be 200, and the purpose of such a setup is to explore how the different penalty functions perform with the increase of dimensions. We would like to construct StackingGroup models by selecting from these models that can handle high-dimensional group-structured data. In order to find the optimal base learner, in the experimental design, the sample size is also fixed to 200, to explore how this Stacking model performs with the increase of dimension p using different base learners, and also to explore the effect of different group sizes on these base learners; through such experimental design, we finally chose to use the three models as the base learners, namely grSubset+grLasso, grLasso, grSCAD as the base learners, and the Stacking model with lasso as the meta-learner is the optimal one, so it is named as StackingGroup; finally, in order to explore the performance of the StackingGroup algorithm proposed in this paper under large-scale data, the experiment is designed to compare the performance of the StackingGroup algorithm with the proposed algorithm with the increase of the sample size and the dimension of p=100, with the increase of the sample size and the dimension of p=100. the algorithm is compared with other models without considering how the algorithm performs as the dimension p increases.

Existing Challenges:

The algorithm proposed in this paper only considers a correlation of 0.5 between the variables in the experimental design phase and does not explore how the model performs in high and low-correlated variables.

**(2)** The paper mentions that the algorithm "outperformed other prediction methods," but it would be more informative if the authors could provide a direct comparison with specific existing methods, including their strengths and weaknesses in different scenarios. This would help the readers understand where StackingGroup excels and where it might fall short.

**[Authors’ Reply]:** Many thanks for your kind help in dealing with our paper. In this revision, In the paper, this paper has been compared with methods that can handle cluster-structured data, see Table 7.

Table 1 Comparison of simulation results of different group structure penalty models in the case of high dimensional large samples

| **Sample Size** | **Model** | ***R*^2^** | ***RMSE*** | ***MAE*** |
| --- | --- | --- | --- | --- |
| *n* = 500 *p* = 100 | grSubset+grLasso | 0.905 | 12.830 | 10.406 |
|  | grLasso | 0.925 | 11.702 | 9.690 |
|  | grSCAD | 0.904 | 11.972 | 9.734 |
|  | grSubset+Ridge | 0.901 | 12.148 | 9.884 |
|  | grMCP | 0.918 | 11.151 | 8.944 |
|  | lasso | 0.921 | 10.947 | 8.870 |
|  | MCP | 0.913 | 11.393 | 9.339 |
|  | SCAD | 0.915 | 11.287 | 9.426 |
|  | alasso | 0.912 | 11.516 | 9.455 |
|  | StackingGroup | **0.919** | **11.020** | **8.947** |
| *n* = 1000 *p* = 100 | grSubset+grLasso | 0.907 | 12.048 | 9.896 |
|  | grLasso | 0.932 | 9.614 | 7.555 |
|  | grSCAD | 0.908 | 10.977 | 8.686 |
|  | grSubset+Ridge | 0.906 | 11.069 | 8.988 |
|  | grMCP | 0.930 | 9.535 | 7.434 |
|  | lasso | 0.929 | 9.558 | 7.490 |
|  | MCP | 0.922 | 10.130 | 8.015 |
|  | SCAD | 0.925 | 9.904 | 7.813 |
|  | alasso | 0.925 | 9.853 | 7.677 |
|  | StackingGroup | **0.932** | **9.399** | **7.318** |
| *n* = 1500 *p* = 100 | grSubset+grLasso | 0.896 | 12.985 | 10.350 |
|  | grLasso | 0.918 | 10.866 | 8.579 |
|  | grSCAD | 0.912 | 10.972 | 8.643 |
|  | grSubset+Ridge | 0.894 | 12.083 | 9.330 |
|  | grMCP | 0.917 | 10.649 | 8.435 |
|  | lasso | 0.915 | 10.800 | 8.587 |
|  | MCP | 0.914 | 10.872 | 8.616 |
|  | SCAD | 0.914 | 10.872 | 8.612 |
|  | alasso | 0.913 | 10.934 | 8.707 |
|  | StackingGroup | **0.918** | **10.642** | **8.437** |
| *n* = 2000 *p* = 100 | grSubset+grLasso | 0.899 | 13.133 | 10.350 |
|  | grLasso | 0.925 | 10.829 | 8.664 |
|  | grSCAD | 0.922 | 10.934 | 8.695 |
|  | grSubset+Ridge | 0.899 | 12.437 | 9.815 |
|  | grMCP | 0.925 | 10.677 | 8.512 |
|  | lasso | 0.924 | 10.737 | 8.516 |
|  | MCP | 0.924 | 10.801 | 8.584 |
|  | SCAD | 0.925 | 10.703 | 8.509 |
|  | alasso | 0.923 | 10.817 | 8.612 |
|  | StackingGroup | **0.925** | **10.667** | **8.478** |

In the above table, we discussed how the StackingGroup algorithm proposed in this paper compares with algorithms that can handle high-dimensional group-structured data, but only discussed how well these models work as the sample size increases, and did not discuss how well these models work as the dimensionality increases if the sample size is controlled? This is our shortcoming, and we also hope to use the algorithm to deal with more practical problems in the next practice.

**(3)** In terms of experimental evaluation, the paper mainly focuses on statistical metrics (*R^2^*, *RMSE*, *MAE*). It would be valuable to provide additional context or case studies to illustrate how the algorithm's enhanced predictive accuracy can lead to meaningful insights or applications in various domains. Real-life use cases could strengthen the practical relevance of the algorithm.

**[Authors’ Reply]:** Many thanks for your kind help in dealing with our paper. In this revision, The background knowledge about the evaluation metrics of *R^2^*, *RMSE*, and *MAE* has been replied to by the first reviewer; in the paper, for the evaluation of the model we chose *R^2^*, *RMSE*, and *MAE* to reflect the performance of the model from different perspectives, *R^2^* measures how well the model fits the observed data, *RMSE* measures the root-mean-square error between predicted and observed values, and MAE measures the mean absolute error between predicted and observed values. mean absolute error between the predicted values and the observed values, by considering these three evaluation indexes, the fit and prediction ability of the model can be evaluated more comprehensively; if only one index is used to evaluate the model, it will make the experimental results biased, and the use of the three evaluation indexes at the same time can serve to corroborate each other and assist each other, for example, in the model evaluation, the *R^2^* is higher but the *MAE* and the *RMSE* are larger, which suggests that the model may be better in fitting the data but have a large error in prediction accuracy, while a lower *R^2^* but smaller *MAE* and *RMSE* may indicate that the model is worse in fitting the data but higher in prediction accuracy. Therefore, using these three evaluation methods can assess the performance of the model more comprehensively and objectively, and also provide a direction for the application of the model. In Table 7 of the paper, it can be observed that the StackingGroup algorithm is either equal or optimal in terms of model fit compared to other algorithms, and the algorithm performs the best in terms of prediction, although the difference is not very large, the algorithm has certain advantages compared to other algorithms, especially for the problem of variable selection of high-dimensional group structure data. Of course, due to the short time, the algorithm can not be applied to financial and genomic data sets to reflect, but in the subsequent research, we will be committed to the optimization of the algorithm and the application of the scenarios, looking forward to more of its performance.

**(4)** The paper's clarity is commendable, but a more detailed explanation of the algorithm's inner workings, such as its convergence properties, optimization strategies, and computational complexity, would be beneficial. Understanding these aspects can help researchers and practitioners decide when and how to apply the StackingGroup algorithm effectively.

**[Authors’ Reply]:** Many thanks for your kind help in dealing with our paper. In the paper, we propose a new algorithm StackingGroup which works internally as follows:

First, Data preparation phase: the dataset is divided into a training set and a test set, the training set is used to train the base learners, and the test set is used to validate and evaluate the model performance.

Second, base learner training phase: the training set is fed into three base learners, including grSubset+grLasso, grLasso, and grSCAD, and finally the models of the three base learners are obtained.

Third, Base Learner Prediction Phase: input the training set into the three base learners, get the prediction results of the three base learners, and merge these three prediction results as the new feature inputs of the Meta-model.

Fourth, the Meta-learner training phase: uses the Lasso model as a Meta-learner for training, and predicts the actual results of the test set that was not previously involved in training by the new features and actual results of the training set.

Fifth, Meta-learner testing phase: use the test set input to the base learner to get the prediction results of the three models, then combine the three prediction results as the new feature input to the meta-model, filter the features according to the dynamic threshold to get the final prediction results.

The convergence of the StackingGroup algorithm mainly depends on the convergence of the base learners and the convergence of the meta-learner. Since all three base learners are based on the L1 regular model, their convergence is relatively good. While the meta-learner is trained using the Lasso model, the model convergence can also be improved by adjusting the hyperparameters.

The optimization strategy of the StackingGroup algorithm is by choosing some base learners with better performance, and also need to pay attention to avoid overfitting problems.

The computational complexity of the StackingGroup algorithm mainly depends on the training time of the base learner and the training time of the meta-learner. In the training process of base learners, the computational complexity of the three base learners is O(*p*^2^*^n^*+*n*^3^), while in the training process of meta-learners, the computational complexity of Lasso is O(*p*^2^*^n^*+*n*^3^), where p is the number of variables and n is the number of samples. Therefore, the total computational complexity of the algorithm will be affected by the number of base learners and the number of samples.

**(5)** It's essential to consider the algorithm's generalizability. Are there specific types of data or situations where it may not perform as well? Discussing the algorithm's limitations and potential failure modes would provide a more balanced view.

**[Authors’ Reply]:** Many thanks for your kind help in dealing with our paper. The StackingGroup algorithm was proposed to solve high dimensional cluster-structured data, therefore, we did not consider how applicable the algorithm is if the data does not have a cluster structure. Thanks for the reviewer's reminder, we will also try how the algorithm performs in datasets that do not have a cluster structure in our subsequent research.

**(6)** The paper could be improved by including visual aids, such as diagrams or charts, to illustrate the StackingGroup algorithm's workflow or the outcomes of the experiments. Visual representations can make complex concepts more accessible.

**[Authors’ Reply]:** Many thanks for your kind help in dealing with our paper. We have briefly described the framework and flow of the algorithm in Figure 2.

Fig 2 Prediction method based on group structure penalty model under Stacking framework

The Stacking framework is as follows:

- The input features are trained for prediction using algorithms such as grSubset+grLasso, grLasso, and grSCAD to aid in feature selection;
- Analyze the error distribution of each algorithm and select the algorithm with larger differences as the layer 1 prediction model. Divide the original dataset and use cross-validation methods to optimize the optimal hyperparameters of each model;
- Produce a new dataset using the partitioned dataset to train the layer 1 prediction algorithm in Stacking separately and output the prediction results;
- Using the newly generated dataset, the layer 2 algorithm in Stacking is trained, based on which the Stacking integrated learning algorithm for multi-model fusion is trained.

**(7)** While the paper provides a strong argument for the StackingGroup algorithm's superior performance, it should also address any overfitting concerns. A discussion of how the algorithm handles overfitting or whether there are cases where it might struggle with noisy data would enhance its credibility.

**[Authors’ Reply]:** Many thanks for your kind help in dealing with our paper. The StackingGroup algorithm is a stacking model with the three models grSubset+grLasso, grLasso, and grSCAD as the base learner and lasso as the meta-learner. Whether we consider it from the base or meta-learner, these models solve the overfitting problem by introducing a regular term, and thus the algorithm consisting of them stacked together is also able to solve the overfitting problem. At the same time, the algorithm is robust to noisy data because it inherits the features and advantages of the base and meta learners. Therefore, reasonable parameter settings and strategies to cope with noisy data are needed, such as data cleaning, missing value, and outlier processing to reduce the interference of noisy data when using the algorithm, so as to use the algorithm for variable selection.

**(8)** Consider discussing the scalability of the algorithm, especially when applied to large datasets. If there are computational limitations or challenges related to handling big data, it's important to acknowledge them.

**[Authors’ Reply]:** Many thanks for your kind help in dealing with our paper. In this revision, The scalability of the algorithm can be initially seen in Table 7 in the paper, as the sample size increases, we can see that the algorithm has a decrease in model fit relative to the scenarios with lower sample sizes, but the decrease is not very large, and the *MAE* and *RMSE* also increase, but the increase is not very large either. Subsequently, we will study the application of the algorithm in ultra-high dimensional cluster datasets based on this in anticipation of its better performance.

**(9)** The paper's conclusion could be strengthened by summarizing the key contributions, highlighting the practical implications of the StackingGroup algorithm, and suggesting potential directions for future research in this area.

**[Authors’ Reply]:** Many thanks for your kind help in dealing with our paper. In this revision,

In the conclusion section, revise as follows:

Variable selection is a hot research issue in the field of statistics, in this paper, we propose a multi-algorithm fusion model (StackingGroup) based on the Stacking integrated learning framework, which is constructed by using grSubset+grLasso, grLasso, and grSCAD as the base learners and lasso as the meta-learners, the algorithm proposed can solve the variable selection problem of high-dimensional group structure data, and it features high recognition of important inter-group variables and important intra-group variables; the effectiveness and stability of the method is verified through numerical simulation and empirical analysis, thus indicating that the algorithm has certain practical application value. However, it still has some limitations, for example, due to the multiple algorithm fusion strategy adopted by the algorithm, the computation of the algorithm changes with the model complexity of the base learner and the meta-learners; algorithm optimization and parallel computation may be required when facing the computation of large-scale data. In our next work, we will continue to investigate how to perform bilayer variable selection and its application to disease prediagnosis and economic forecasting under the Stacking integrated learning framework when the response variable is discrete. In addition, optimization of algorithms, computation, and processing of large-scale datasets are also future research directions in this area.

**(10)** Lastly, the author ensure that all mathematical notations and equations are clear and consistently formatted throughout the paper. Clarity and readability are essential for the paper's success.

**[Authors’ Reply]:** Many thanks for your kind help in dealing with our paper. In this revision, Our team has rechecked the formulas and algorithmic flow, etc. in this paper, and if there are any errors, they have been corrected and red-flagged in the revised manuscript, we would like to thank the reviewers for their reminders and strict requirements.

This formula has been updated in the revised draft:$R^{2}=\frac{\sum_{i=1}^{n} \left(\hat{y}_{i}-\bar{y}\right)^{2}}{\sum_{i=1}^{n} \left(y_{i}-\bar{y}\right)^{2}}=1-\frac{\sum_{i=1}^{n} \left(y_{i}-\hat{y}_{i}\right)^{2}}{\sum_{i=1}^{n} \left(y_{i}-\bar{y}\right)^{2}}$

In the end, we would like to thank for the precious time of the reviewers and editors. We sincerely hope that our point-to-point response and the revised manuscript can address your concerns and satisfy your requirements for publication. We would be grateful if we had the chance to share our work with the readers of **PLOS ONE**.

Sincerely,

Dr.Dongsheng Li

Professor

School of Mathematics and Statistics

Qiannan Normal University for Nationalities

Duyun, China

Email:465164730@qq.com
